# PEPITEM modulates leukocyte trafficking to reduce obesity-induced inflammation

**DOI:** 10.1093/cei/uxad022

**Published:** 2023-03-09

**Authors:** Laleh Pezhman, Sophie J Hopkin, Jenefa Begum, Silke Heising, Daniela Nasteska, Mussarat Wahid, G Ed Rainger, David J Hodson, Asif J Iqbal, Myriam Chimen, Helen M McGettrick

**Affiliations:** Institute of Inflammation and Ageing, University of Birmingham, Birmingham, UK; Institute of Cardiovascular Sciences, University of Birmingham, Birmingham, UK; Institute of Cardiovascular Sciences, University of Birmingham, Birmingham, UK; Institute of Metabolism and Systems Research, University of Birmingham, Birmingham, UK; Institute of Metabolism and Systems Research, University of Birmingham, Birmingham, UK; Institute of Inflammation and Ageing, University of Birmingham, Birmingham, UK; Institute of Cardiovascular Sciences, University of Birmingham, Birmingham, UK; Institute of Metabolism and Systems Research, University of Birmingham, Birmingham, UK; Institute of Cardiovascular Sciences, University of Birmingham, Birmingham, UK; Institute of Inflammation and Ageing, University of Birmingham, Birmingham, UK; Institute of Inflammation and Ageing, University of Birmingham, Birmingham, UK

**Keywords:** obesity, pancreas, T cells, B cells, PEPITEM, inflammation

## Abstract

Dysregulation of leukocyte trafficking, lipid metabolism, and other metabolic processes are the hallmarks that underpin and drive pathology in obesity. Current clinical management targets alternations in lifestyle choices (e.g. exercise, weight loss) to limit the impact of the disease. Crucially, re-gaining control over the pathogenic cellular and molecular processes may offer an alternative, complementary strategy for obese patients. Here we investigate the impact of the immunopeptide, PEPITEM, on pancreas homeostasis and leukocyte trafficking in mice on high-fed obesogenic diet (HFD). Both prophylactic and therapeutic treatment with PEPITEM alleviated the effects of HFD on the pancreas, reducing pancreatic beta cell size. Moreover, PEPITEM treatment also limited T-cell trafficking (CD4^+^ T-cells and KLRG1^+^ CD3^+^ T-cells) to obese visceral, but not subcutaneous, adipose tissue. Similarly, PEPITEM treatment reduced macrophage numbers within the peritoneal cavity of mice on HFD diet at both 6 and 12 weeks. By contrast, PEPITEM therapy elevated numbers of T and B cells were observed in the secondary lymphoid tissues (e.g. spleen and inguinal lymph node) when compared to the untreated HFD controls. Collectively our data highlights the potential for PEPITEM as a novel therapy to combat the systemic low-grade inflammation experienced in obesity and minimize the impact of obesity on pancreatic homeostasis. Thus, offering an alternative strategy to reduce the risk of developing obesity-related co-morbidities, such as type 2 diabetes mellitus, in individuals at high risk and struggling to control their weight through lifestyle modifications.

## Teaser

In this manuscript, we demonstrate for the first time that therapeutic administration of the immunopeptide, PEPITEM, limits the pathological impact of obesity on the pancreas and systemic leukocyte trafficking, dampening the effects of obesity-induced systemic low-grade inflammation. Further work is required to determine the utility of PEPITEM as an alternative strategy to reduce the risk of developing obesity-related co-morbidities, such as type-2 diabetes, in individuals at high risk and struggling to control their weight through lifestyle modifications.

## Graphical Abstract

**Figure:**
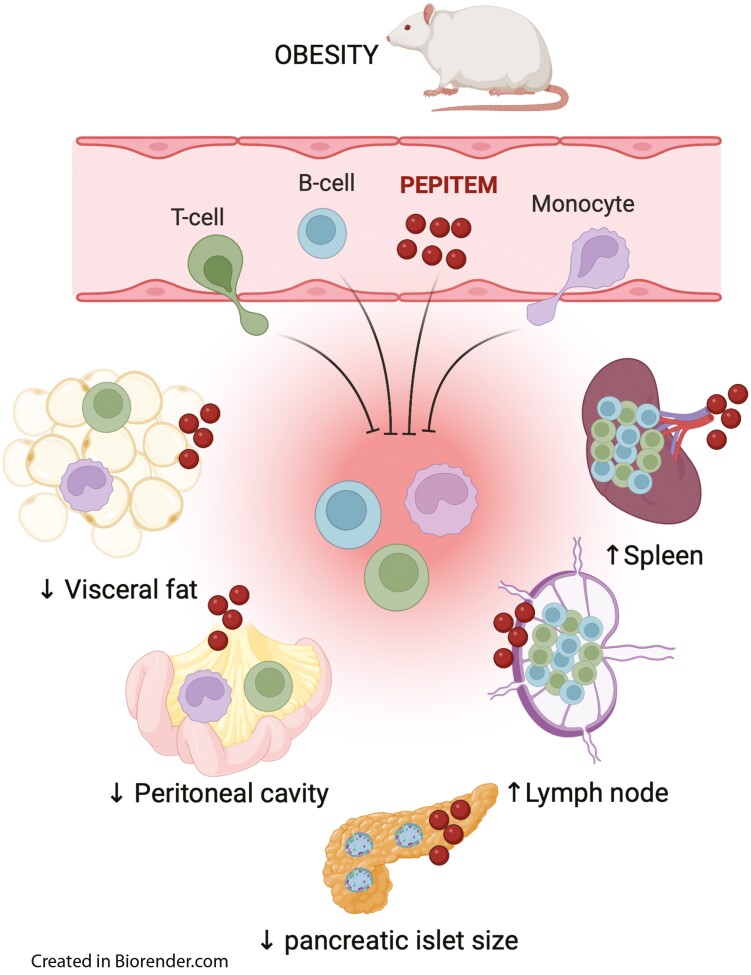

## Introduction

Obesity is characterized by pathogenic changes in adipose tissues leading to low-grade systemic chronic inflammation and aberrant accumulation of pro-inflammatory leukocytes within adipose tissues. Indeed, obese individuals have increased risk of developing co-morbidities, such as type 2 diabetes mellitus (T2DM) [1], hepatic steatosis, and cardiovascular disease, leading to higher morbidity and mortality rates compared to lean individuals. The estimated number of individuals who are deemed obese is raising at an alarming rate [2] with an expected 1 billion adults and 206 million children worldwide predicted to be clinically obese by 2025, making obesity and obesity-related diseases the major global health challenge. This is coupled with the substantial economic impact of these diseases, estimated as 2.19% of global gross domestic product or ~US$1900 billion globally in 2019 [3]. Despite this, we still understand little about how the inflammation associated with obesity drives pathology. Moreover, current clinical management often targets alterations in lifestyle choices (e.g. exercise and weight loss) or more extreme surgical interventions.

Obesity induces a dramatic transformation in the composition of leukocytes resident in the adipose tissue, which experiences an influx of pro-inflammatory leukocytes [4, 5] and detrimental changes in the homeostatic regulatory populations (T_reg_, NK cells, and ILCs), including loss in numbers and/or the acquisition of pathogenic functions [6–8]. The accumulation of pro-inflammatory macrophages (so-called M1 macrophages) is widely accepted to play a central role in obesity-induced pathology [4]. However, studies suggest that the recruitment of CD8^+^ T-cells precedes, and potentially drives, the infiltration of M1 macrophages in murine models of obesity [5, 9]. Furthermore, intravital microscopy showed augmented leukocyte adhesion both locally in visceral adipose tissue (VAT) [10] and systemically in the cremaster muscle [2] in obese mice when compared to non-obese controls. Surprisingly, obesity did not alter recruitment to subcutaneous sites in these animals [8], indicating that different adipose tissue depots may differentially regulate leukocyte recruitment in obesity.

Obese adipose tissue also exhibits dysregulation in metabolic processes involved in insulin sensitivity, leading to pancreatic beta-cell damage, insulin resistance, and eventually hyperglycaemia that underpins T2DM [11]. Indeed, T2DM is characterized by increased number and size of beta cells [12] resulting in functional insufficiency. Adiponectin is an adipose-derived adipokine that functions as insulin-sensitizing hormone and anti-inflammatory cytokine [13]. It has been shown to negatively regulate adhesion molecule expression on blood vascular endothelial cells [14] to limit leukocyte recruitment [15]. We have shown that adiponectin stimulates the release of an immunopeptide (PEPITEM) from B cells, limiting T-cell migration into inflamed tissues [16]. However, the adiponectin-PEPTIEM pathway is dysregulated in patients with T1DM and rheumatoid arthritis, due to reduced ability to respond to adiponectin [16]. Furthermore, groups have now shown the therapeutic potential of synthetic PEPITEM in murine models of immune-mediated inflammatory diseases (IMIDs) [16, 17]. As the circulating levels of adiponectin are significantly decreased in patients with obesity [18] and T2DM [19], there is a distinct possibility that these individuals are unable to generate sufficient PEPITEM and therefore would benefit from replacement therapy. Using obesogenic-dietary preclinical models, we have investigated the therapeutic efficacy of PEPITEM to limit pancreatic beta-cell damage and modulate systemic leukocyte trafficking.

## Methods

### Mouse models

Eight-week-old, male, C57Bl/6J wild type (WT) mice were purchased from Charles River and were maintained in a specific pathogen free facility, with free access to food. Environmental conditions were: 21 ± 2°C, 55 ± 10% relative humidity, and a 12 h light-dark cycle. Mice were fed high fat diet (HFD) containing 60% fat (cat no. D12492, Research Diets, INC) for up to 12 weeks. Alzet mini pumps 2006 (Charles River) containing 3.5 mg/ml of PEPITEM (SVTEQGAELSNEER-PEG (352)-Amide; Cambridge Research Biochemicals Limited; Cambridge, UK) or phosphate buffered saline (PBS) were implanted at baseline (prophylactic administration) or following 6 weeks of HFD (therapeutic administration), continuously releasing 0.822 mg of content/week. Body weight was assessed weekly. Intraperitoneal glucose tolerance tests (IPGTT) were assessed at 3, 6, 8, and 12 weeks in mmol/l using Contour XT glucometer as previously described [20]. Mice were culled by cardiac puncture with blood collected into EDTA-coated Eppendorfs. The peritoneal cavity was lavaged with 5 mM EDTA, spleen and inguinal lymph nodes were isolated and stored in PBS. Gonadal visceral fat pads and abdominal subcutaneous fat were stored in RPMI 1640 media (Gibco) containing 2% BSA and the pancreas was FFPE as previously described [20].

### Sample processing for flow cytometry analysis

All tissue samples were weighed prior to processing. Fat tissue was broken into pieces, and incubated in an enzyme cocktail consisting of 200 μg/ml Collagenase P (Sigma), 800 μg/ml Collagenase Dispase, and 100 μg/ml DNase (both Merck) diluted in RPMI containing 2% FBS at 37°C for 30 min before being passed through 70 μM filter. Samples were washed in RPMI containing 2% FBS at 400*g* for 10 min and the stromal vascular fraction re-suspended in RBC lysis buffer for 10 min. Samples were then washed by centrifugation at 400*g* and re-suspended in RPMI containing 2% FBS. The spleen and lymph node were crushed through a 40 μM filter. Spleen, lymph node, and blood samples were all incubated in the RBC lysis buffer as described prior to re-suspension in MACS buffer. Peritoneal lavage fluid (PLF) was centrifuged at 400*g* for 5 min and the cells were resuspended in MACS buffer.

All samples were blocked with FcR blocker (Miltenyi Biotec) prior to staining with the following antibodies and Zombie Aqua (Biolegend) for 20 min at 4°C prior to washing and fixation with 2% PFA: anti-CD45.2 BV605 (clone 104; Biolegend), anti-CD3 PECy7 (clone 145-2c11), anti-CD4 eFluor450 (clone GK1.5), anti-CD8 PE-TexasRed (clone 5H10), anti-CD44 FITC (clone IM7), anti-CD25 AF700 (clone PC61.5), anti-KLRG1 APC-eFluor780 (clone 2F1), anti-CD62L PE (clone MEL-14), anti-CD19-APC (clone 1D3), anti-CD45 APC-CY7 (clone 104), F4/80 FITC (clone BM8), anti-CD11c PE-Cy7 (clone N418), anti-Gp38 PE (clone 8.1.1; all from Thermofisher), anti-CD23 BV421 (clone B3B4), anti-CD93 BV650 (clone AA4.1), anti-CD43 PerCp-Cy5.5 (clone S7), anti-CD21/35 PE (clone 7G6), anti-Siglec F TexasRed (clone E50-2440), and Ly6G APC (clone 1A8; all from BD). Compensation controls were generated using the cells isolated from the spleen. Immediately prior to analysis, CountBright beads (Invitrogen) were added and samples were acquired using Fortessa-X20 and data were analysed offline using FlowJo (V-10.2.6) (Supplementary Fig. S1).

### Immunofluorescence imaging

FFPE pancreas tissue sections 5 μm in depth were subjected to deparaffinization using histoclear (Sigma) followed by incubation in decreasing concentrations of ethanol prior to antigen retrieval using a 10 mM citrate buffer at pH6 for 25 minutes [20]. Slides were blocked using 0.1% triton X-100 in 2% BSA for 1 h, prior to incubation at 4^o^C overnight with anti-insulin (1:500, clone C27C9, Cell Signalling) and anti-glucagon (1:2000, clone K79bB10, Sigma) antibodies diluted in blocking solution. Following 3 × 10-min washes in blocking solution, slides were incubated for 2 h in the dark at room temperature with goat-anti-mouse IgG AF488 and goat-anti-rabbit IgG AF633 (both Thermofisher) diluted in blocking solution. Slides were washed as described and mounted using Vectrashield Mounting Media with DAPI and stored at 4^o^C.

Slides were imaged using Zeiss Axioscan 7 Slide Scanner at 20× magnification using the same acquisition parameters for each slide. Images were initially processed with ZEN Software (Black Edition v2.2) into digital arrays, which were subsequently analysed with ImageJ (1.53K). Insulin and glucagon were used to identify beta and alpha cells, respectively and DAPI to identify total nuclei (cell number). Average number of islets and islet area were determined and expressed as number or area/10 000 μm^2^ of the whole pancreatic section area, as previously described [12].

### Statistical analysis

Data were analysed using GraphPad Prism and presented as mean ± SEM for *n* independent experiments. Normality was assessed using Shapiro–Wilk test. Univariate analysis was performed using unpaired *t*-test. *P* < 0.05 was deemed statistically significant.

## Results

**Figure 1:**
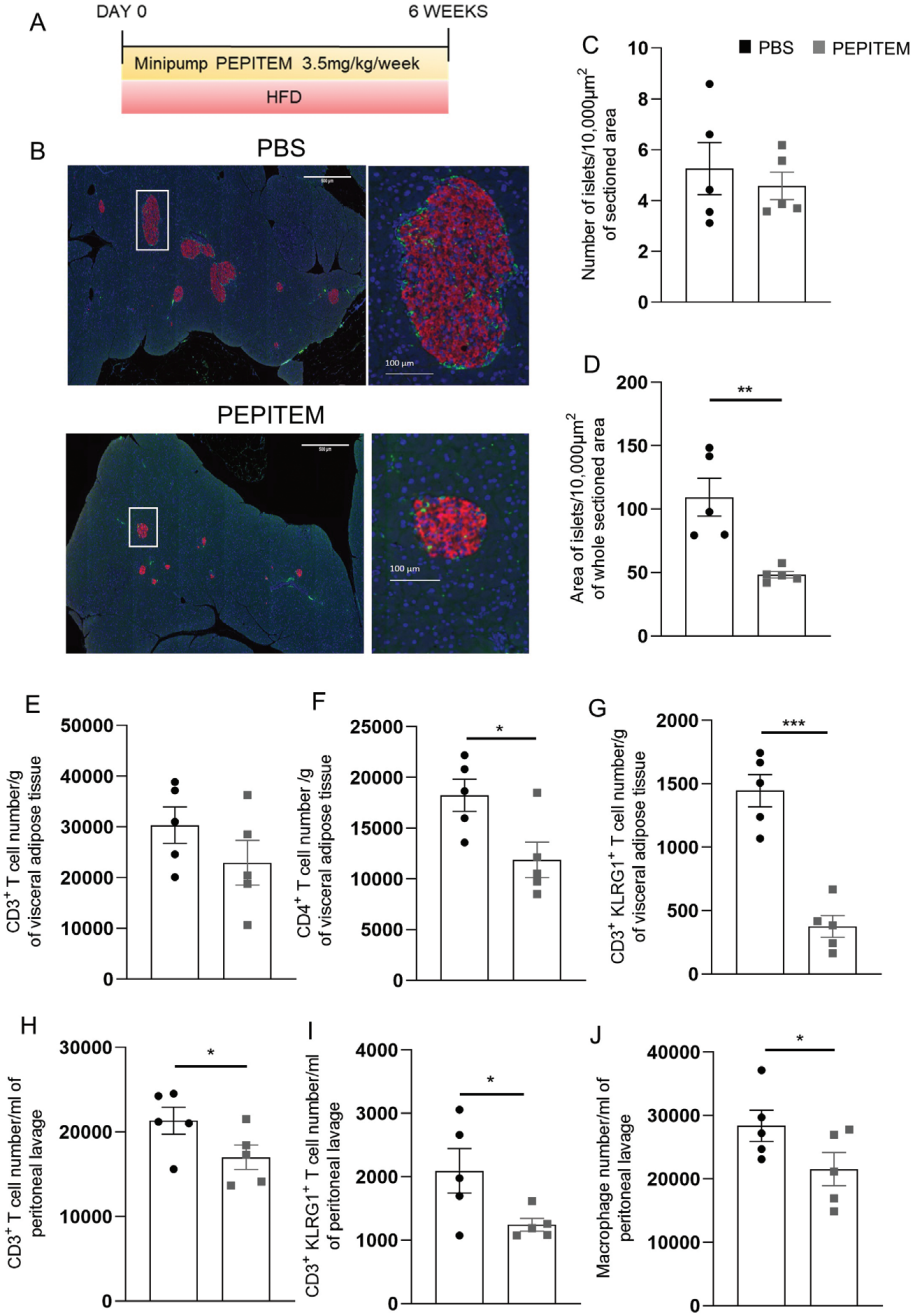
Prophylactic treatment with PEPITEM reduced leukocyte trafficking induced by 6 weeks of a high-fat obesogenic diet. (**A**) Schematic representation of experimental time course where wild-type male mice were prophylactically implanted with a mini pump, which continuously released 0.0822 mg/week of PEPITEM or PBS (as a control), and fed HFD for 6 weeks. **(B)** Representative images of pancreatic islets from PBS or PEPITEM-treated mice were stained with insulin-producing beta cells in red and glucagon-releasing alpha cells in green and imaged (scale bar = mm^2^) (for colour figure refer to online version). White box represents a single pancreatic islet shown at increased magnification (scale bar = µm^2^). Images were quantified for **(C)** the number of the islets/10 000 µm^2^ of whole area and **(D)** the area of islets/10 000 µm^2^ of whole area. **(E)** visceral adipose tissue CD3^+^ T-cells, **(F)** visceral adipose tissue CD4^+^ T cells, **(G)** visceral adipose tissue CD3^+^KLRG1^+^ T-cells, **(H)** peritoneal lavage CD3^+^ T cells, **(I)** peritoneal lavage CD3^+^KLRG1^+^ T cells and **(J)** peritoneal lavage F4/80^H^CD11c^Int^ macrophages were quantified using flow cytometry analysis. Absolute number of immune cells were normalized to bead counts and plotted as number per g of adipose tissue and per ml of peritoneal lavage. Data are mean ± SEM using *n* = 5 mice per group from *n* = 1 independent experiment. *P < 0.05, **P < 0.01, and ***P < 0.001 by unpaired *t*-test compared to PBS treated.

Obesity induces systemic metabolic and immunological changes driving various metabolic syndromes and immune-mediated inflammatory diseases (IMIDs). Initially, we examined whether prophylactic administration of the immunopeptide, PEPITEM, modulated obesogenic diet inducing inflammation over a 6-week period (Fig. 1A**).** As expected body weight increased with the HFD over this timeframe and was unaffected by PEPITEM-treatment (Supplementary Fig. S2A). Similarly, PEPITEM had no effect on fasting glucose tolerance or insulin resistance at either week 3 or 6 of HFD (Supplementary Fig. S2B). As pancreatic beta-cell mass is a key pathological feature in both obesity and T2DM [21], we assessed the effect of PEPITEM on the number and size of pancreatic islets following 6 weeks of HFD (Fig. 1B). Prophylactic PEPITEM treatment had no effect on the number of pancreatic islets (Fig. 1C), but significantly reduced their individual area when compared to PBS treated mice on HFD (Fig. 1D**),** indicating that PEPTIEM was able to inhibit the dietary expansion of pancreatic islets.

Obesity and T2DM induce systemic low-grade chronic inflammation that is known to influence leukocyte composition, thus their trafficking profiles, within peripheral tissues. Significantly fewer T-cells, in particular KLRG1^+^ CD3^+^ T-cells, were found in the VAT (Fig. 1E–G) and within the peritoneal cavity (Fig. 1H–I) in mice treated prophylactically with PEPITEM compared to the HFD control group. We also observed a significant reduction of macrophages within the peritoneal cavity of PEPITEM-treated animals compared to PBS (Fig. 1J) back to levels seen at in chow-fed 12-week-old mice (23 267 ± 2927 mean ± SEM macrophages/ml, *n* = 5). These data strongly indicated that PEPITEM is able to block the effects of an obesogenic diet on T-cell and macrophage trafficking. Of note, prophylactic PEPITEM treatment had no effect on the number of T-cell subsets, B-cell subsets, neutrophils, eosinophils, or macrophages in the blood, inguinal lymph node, or spleen (Supplementary Table S1).

**Figure 2:**
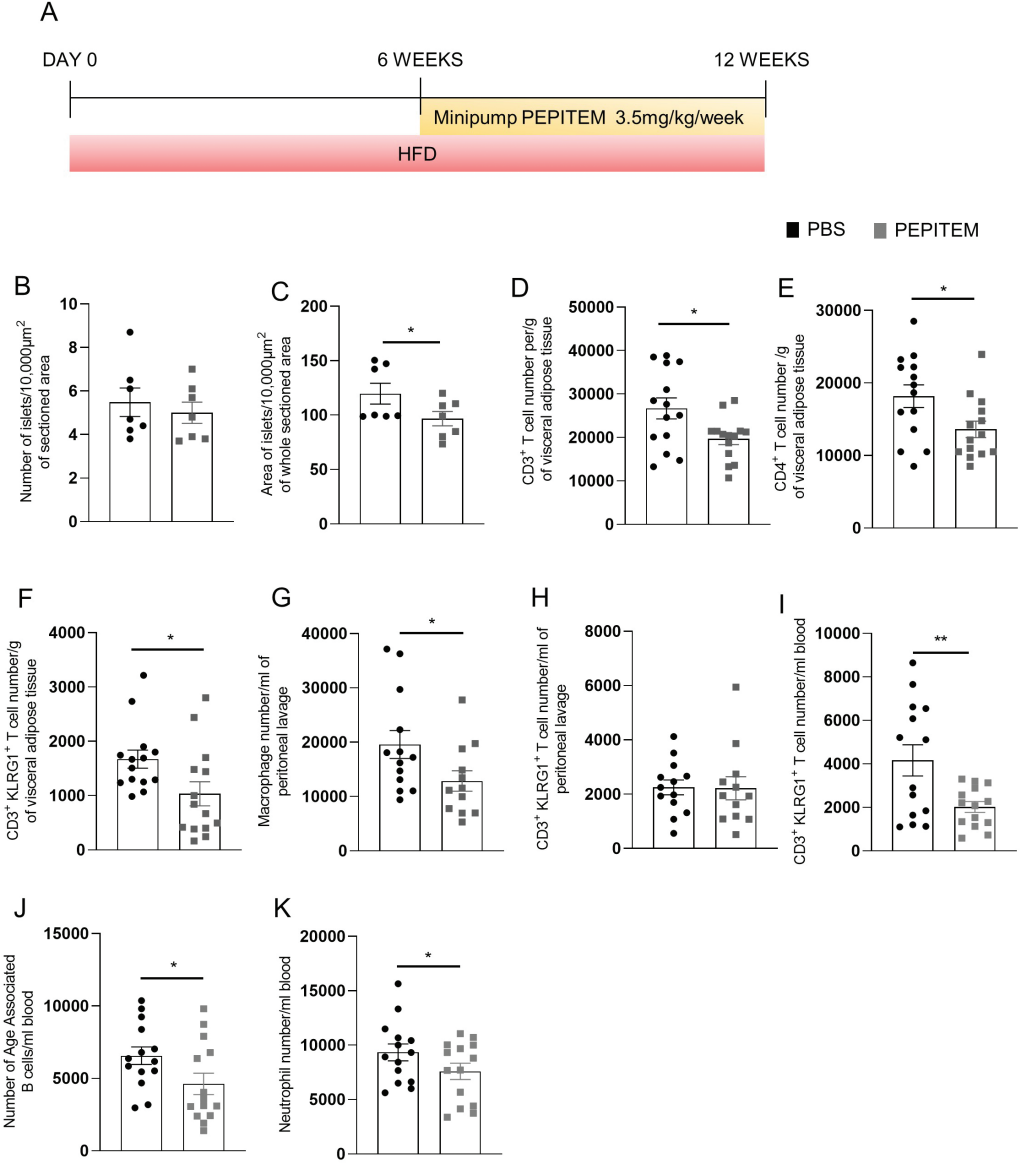
Therapeutic treatment with PEPITEM altered the patterns of leukocyte trafficking into peripheral tissues induced by 12 weeks of a high-fat obesogenic diet. (**A**) Schematic representation of experimental time course where mice were fed HFD for 12 weeks. At week 6, mice were implanted with a mini pump to allow therapeutic continuous release 0.0822 mg/week of PEPITEM or PBS (as a control) for the last 6 weeks of the HFD. Pancreatic islets from PBS or PEPITEM-treated mice were stained insulin-producing beta cells and glucagon-releasing alpha cells. Images quantified for **(B)** the number of the islets/10 000 µm^2^ of whole area and **(C)** the area of islets/10 000 µm^2^ of whole area. **(D)** Visceral adipose tissue CD3^+^ T cells, **(E)** visceral adipose tissue CD4^+^ T cells, **(F)** visceral adipose tissue CD3^+^KLRG1^+^ T-cells, **(G)** peritoneal lavage F4/80^H^CD11c^Int^ macrophages, **(H)** peritoneal lavage CD3^+^KLRG1^+^ T cells, **(I)** blood CD3^+^KLRG1^+^ T-cells, **(J)** blood CD19^+^CD43^+^CD93^+^CD23^−-^ CD21^−^ age-associated B-cells, and **(K)** blood LyG6^+^ neutrophils were quantified using flow cytometry analysis. Absolute number of immune cells were normalized to bead counts and plotted as number per g of adipose tissue and per ml of peritoneal lavage or blood. Data are mean ± SEM using *n* = 14 mice per group from *n* = 1 independent experiment. *P < 0.05 and **P < 0.01 by unpaired *t*-test compared to PBS treated.

**Figure 3:**
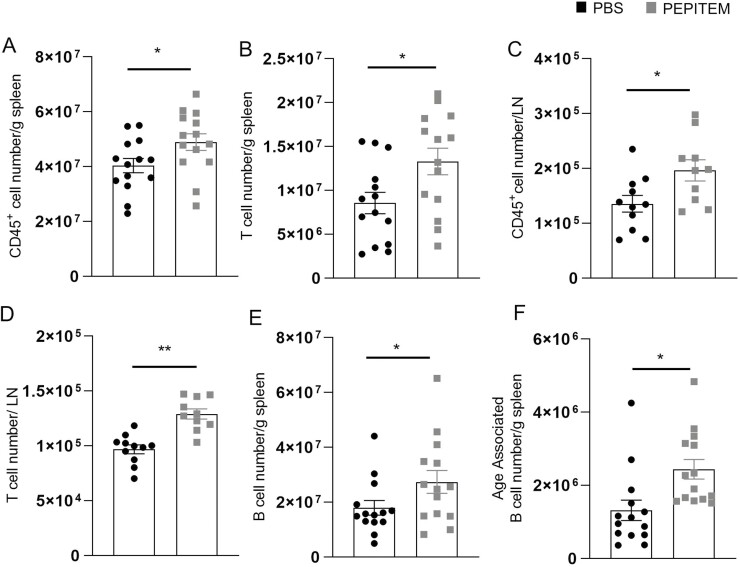
Therapeutic treatment with PEPITEM altered leukocyte trafficking into secondary lymphoid tissues in obese mice. Mice were fed HFD for 12 weeks and received 0.0822 mg/week of PEPITEM or PBS as a control for the last 6 weeks of the HFD for the full duration. **(A)** Splenic CD45^+^ T cells, **(B)** splenic CD3^+^ T cells, **(C)** inguinal lymph node CD45^+^ T cells, **(D)** inguinal lymph node CD3^+^ T cells, **(E)** splenic CD19^+^ B cells, and **(F)** splenic age-associated B-cells were quantified using flow cytometry analysis. Absolute number of immune cells were normalized to bead counts and plotted as number per g of tissue. Data are mean ± SEM using *n* = 14 mice per group from *n* = 1 independent experiment. *P < 0.05 and **P < 0.01 by unpaired *t*-test compared to PBS treated.

Next, we investigated the therapeutic potential of PEPITEM to reverse chronic inflammation and the development of T2DM/obese pancreas by feeding mice HFD prior to implantation of the PEPITEM slow-release pumps. As observed in the 6-week model, PEPITEM had no effect on the weight of the mouse or glucose tolerance at either 8 or 12 weeks of diet (Supplementary Fig. 2C and D). Excitingly, therapeutic administration of PEPITEM (Fig. 2A) significantly reduced the pancreatic islet size, without affecting the overall number of islets, when compared to control mice (Fig. 2B and C). Thus, indicating that PEPITEM therapy can overcome/reverse diet induced increase in beta-cell area. Moreover, PEPITEM dramatically altered the composition of leukocyte subsets in the blood, inguinal lymph node, spleen, VAT, and peritoneal cavity (Fig. 2D–K), but had no effect on subcutaneous adipose tissue (data not shown). Similar to the 6-week HFD model, we observed a significant reduction in the number of CD3^+^ T-cells (Fig. 2D), CD4^+^ T-cells (Fig. 2E), CD3^+^KLRG1^+^ T-cells (Fig. 2F) in the VAT and in macrophage numbers in the peritoneal cavity (Fig. 2G) at 12 weeks of HFD following PEPITEM therapy compared to mice treated with vehicle control. Unlike the prophylactic study, we observed no effect of therapeutic PEPITEM on CD3^+^KLRG1^+^ T-cells numbers in the peritoneal cavity (Fig. 2H) following 12 weeks of HFD. The number of CD3^+^KLRG1^+^ T-cells (Fig. 2I), age-associated B-cells (Fig. 2J), and neutrophils (Fig. 2K) were also lower in the blood of PEPITEM-treated mice. By contrast, PEPITEM therapy increased the number of CD45^+^ leukocytes, in particular CD3^+^ T-cells, in the spleen (Fig. 3A and B) and inguinal lymph node (Fig. 3C and D) compared to control animals. B cell (Fig. 3E) and age-associated B-cell (Fig. 3F) numbers were also found to be elevated in the spleen of PEPITEM-treated mice compared to PBS-treated mice. Thus, PEPITEM therapy systemically modulates leukocyte trafficking through the VAT, peritoneal cavity, and secondary lymphoid organs (SLO), potentially reversing the pathologic effects of the obesogenic diet on these tissues.

## Discussion

Obesity drives systemic low-grade inflammation, altering metabolic, and immune processes, and leading to numerous co-morbidities, including T2DM. Here we report for the first time that the immunopeptide PEPITEM is able to reverse the effects of an obesogenic diet on (i) pancreatic beta-cell size, (ii) aberrant T-cell trafficking into VAT, and (iii) leukocyte mobilization from SLO.

Obesity has been reported to induce pancreatic beta cell mass expansion in obese non-diabetic patients [22–24] and also mice fed on HFD for 8 weeks [25, 26]. The mechanism regulating this expansion is heavily debated within the literature, with groups postulating enhanced proliferation/regeneration of beta cells [22, 25] and others indicating that the changes in mass are not linked with the proliferation or apoptosis of these cells [24, 26]. Recently, 14-3-3 family members (14-3-3ξ is the parent protein of PEPITEM) have been postulated to be essential for pancreatic beta cell expansion; with significantly higher levels of proliferation observed *in vitro* when murine or human islet beta cells were cultured in the presence of a pan 14-3-3 inhibitor [27]. In all cases, studies reported a degree of beta cell dysfunction occurs and that this is likely to lead eventually to glucose intolerance and insulin resistance associated with T2DM. Interestingly, PEPITEM treatment, either prophylactically or therapeutically, alleviated the effects of HFD on the pancreas, reducing pancreatic beta cell size. Similar observations have been reported in obese mice treated prophylactically with metformin for 8 weeks, although the exact mechanisms behind this were not defined [26]. Of note, PEPITEM had no effect on obesogenic diet induced weight gain and therefore lipid storage in adipose tissues. Collectively these studies indicate the potential for certain clinical interventions to uncouple obesity-induced changes in pancreatic homeostasis from those associated with lipid metabolism/storage in the adipose tissue and weight gain—thus potentially offering an alternative means to reduce the risk of developing T2DM-associated pancreatic damage in individuals at high risk.

In addition, obesity has been reported to alter the trafficking profiles of T-cells, where memory CD4^+^ T-cells from HFD-fed mice preferentially migrate to non-lymphoid (i.e. peripheral tissues) in a CXCR3-dependent manner, even in chow-fed mice [28]. Similarly, others have reported elevated numbers of senescent or senescent-associated (CD153^+^) T-cells within the VAT of obese mice following 16 and 18 weeks of HFD diet [29, 30]. Indeed, senescent-associated CD153^+^CD4^+^ T-cells from obese mice have been reported to drive metabolic dysregulation and inflammation within the VAT—where their adoptive transfer into non-obese mice induced insulin resistance and pro-inflammatory cytokine production in the recipient mice [30]. Likewise, greater numbers of senescent CD8^+^CD57^+^ T-cells were detected in the omental tissue of pre-diabetic or T2DM patients with BMIs in the overweight, rather than obese range, compared to normoglycemia controls [31]. Here we report for the first time that PEPITEM treatment, either prophylactically or therapeutically, limits T-cell trafficking (CD4^+^ T-cells, CD3^+^KLRG1^+^ senescent [32, 33] T-cells) to obese VAT. This agrees with previous data demonstrating that prophylactic treatment with PEPITEM reduces CD3^+^ T-cell trafficking in numerous models of inflammation, including zymosan-induced peritonitis; ischemia-reperfusion injury in the liver; salmonella infection of the liver; virally induced Sjogren’s Syndrome [16]; and MLR/lpr induced lupus nephritis [17]. Crucially levels of CD3^+^KLRG1^+^ senescent T-cells are also reduced in obese VAT following either prophylactic or therapeutic treatment, indicating that their production and/or trafficking is influenced by PEPITEM. It is quite possible that PEPITEM is able to reverse the metabolic effects of HFD and restore normal trafficking patterns between peripheral and lymphoid tissues.

Whilst trafficking through secondary lymphoid organs is not routinely examined in pre-clinical models of obesity, there are a few studies describing alterations in leukocyte numbers within these tissues, which in some cases have been attributed to pathological changes in the barrier function of the lymphatics and abnormal lymph node architecture [34]. For example, T-cell migration into the mesenteric lymph node [4, 35] and dendritic cell migration into local draining lymph nodes [4, 35] were reduced in obese mice compared to chow-diet controls. Moreover, loss of CCL21 gradients resulted in aberrant lymph node follicle organization in obese mice, leading to a reduction in CD4^+^ and CD8^+^ T-cell numbers within the SLO [36]. Our data suggest that PEPITEM therapy potentially reverses these pathogenic effects, as elevated numbers of T and B cells were observed in the SLO analysed here when compared to the untreated HFD controls. Note these changes were not observed following prophylactic PEPITEM administration over 6 weeks. Altered trafficking in response to PEPITEM therapy could be due to increased trafficking into SLO or by retention of cells within these organs. Indeed, PEPITEM is known to mediate it effects through sphingosine-1-phosphate (S1P) [16], which has previously been demonstrated to be a potent inhibitor of lymphocyte migration across high endothelial venules (HEV, i.e. entry into lymph nodes [37]) and lymphatic endothelium (i.e. exit from lymph nodes [38]). Combined with the fact that some populations show concomitant decreases within the circulation, this argues for PEPITEM-induced S1P modulating influx across HEV into the SLO rather than retaining cells within these structures. That said, further tracking studies are required to ascertain the exact mechanisms by which PEPITEM influences the composition of SLO during obesity.

Numerous studies have reported an influx of monocytes into VAT at the early stages of HFD-induced obesity [4] and T2DM [1], where they differentiate into M1 macrophages to drive pro-inflammatory responses. Whilst PEPITEM did not influence the absolute numbers of macrophages in the VAT, it would be interesting to further characterize their phenotype to ascertain whether PEPITEM alters the balance between M1 and M2 macrophages within this tissue. Others have reported that PEPITEM treatment reduced F4/80^+^ macrophage numbers in the kidneys of mice with glomerular nephritis [17], indicating PEPITEM can influence the trafficking of other cell types beyond T-cells as originally reported [16]. Interestingly, PEPITEM treatment altered macrophage numbers in the peritoneal cavity at both 6 and 12 weeks, where fewer macrophages were observed. This could be due to reduced trafficking of monocytes into the cavity or enhanced exit of macrophages, potentially by trafficking to the other neighbouring tissues, such as the omentum as has recently been described [39]. To the best of our knowledge, no groups have looked at macrophage numbers or phenotype in the peritoneal cavity in mice on HFD, making it difficult to critique this observation.

Obesity has long been treated with lifestyle interventions (low-calorie diet, and increase in exercise) followed by bariatric surgery in extreme cases. Five drugs are currently approved for use in the USA/Europe (e.g. orlistat—limits fat absorption; or lorcaserin—appetite suppressant), however, whilst these provide a valuable alternative option they often offer only moderate weight loss over to standard diet or exercise regimes (reviewed by [40]). In addition, a number of studies are investigating the benefits of therapeutic targeting of leukocyte trafficking in obesity-related inflammatory diseases, including T2DM (reviewed by [1]), however, many of these immunomodulatory therapies only show partial reduction in disease pathogenesis. As an endogenous molecule (akin to insulin), therapeutic administration of PEPITEM may provide the opportunity to re-establish control over both local and systemic metabolic and inflammatory processes underlying the pathogenesis of obesity.

To conclude, therapeutic administration of the immunopeptide, PEPITEM, limits the pathological impact of obesity on the pancreas and systemic leukocyte trafficking, dampening the effects of obesity-induced systemic low-grade inflammation. Importantly, we reveal for the first time that the actions of PEPITEM uncouple obesity-induced pathogenic lipid storage and metabolism in the adipose tissue (weight gain) from the systemic effects of obesity on pancreas homeostasis. Further research is now needed to fully elucidate how PEPITEM regulates beta-cell area and function, and whether this is uncoupled from lipid storage. Moreover, further tracking studies are required to ascertain the exact mechanisms by which PEPITEM influences the composition of the peritoneal cavity and SLO during obesity (regulation of entry, retention, or exit) and the impact this has on immune responses. Collectively our data highlights the potential for PEPITEM as a novel therapy to combat the systemic low-grade inflammation experienced in obesity and minimize the impact of obesity on pancreatic homeostasis. Thus, offering an alternative strategy to reduce the risk of developing obesity-related co-morbidities, such as T2DM, in individuals at high risk and struggling to control their weight through lifestyle modifications.

## Supplementary Material

uxad022_suppl_Supplementary_Material

## Data Availability

All data related to this study is included within the manuscript at point of publication.

## Abbreviations

ABC: age-associated B cells
FBS: foetal bovine serum
FFPE: formalin-fixed paraffin-embedded
HFD: high fat diet
ILCs: innate lymphoid cells
iLN: inguinal lymph node
IMIDs: immune-mediated inflammatory diseases
IPGTT: intraperitoneal glucose tolerance tests
KLRG1: killer cell lectin-like receptor subfamily G member 1
NK: natural killer cells
PBS: phosphate buffered saline
PEPITEM: peptide inhibitor of trans-endothelial migration
PLF: peritoneal lavage fluid
RBC: red blood cell
S1P: sphingosine-1-phosphate
SLO: secondary lymphoid organs
T reg: regulatory T cells
T2DM: type 2 diabetes mellitus
VAT: visceral adipose tissue
WT: wild type
