# Supplementary material for: PEPITEM modulates leukocyte trafficking to reduce obesity-induced inflammation
Source: Clin Exp Immunol. 2023 Mar 9;212(1):1–10. doi: 10.1093/cei/uxad022 (PMC10081110; doi:10.1093/cei/uxad022)
Supplement: uxad022_suppl_Supplementary_Material [file uxad022_suppl_supplementary_material.docx]

**Supplementary Figure 1 – Flow cytometry gating strategy.** Leukocytes were identified using FS/SS, doublets removed using pulse width vs FS and dead cells excluded using zombie aqua. Subsequently CD45^+^ leukocytes were gated from which the major subsets of cells were identified: CD3^+^ T-cells, CD3^+^CD4^+^ T-cells, CD3^+^CD8^+^ T-cells, CD3^+^KLRG1^+^ T-cells; CD19^+^ B-cells, CD19^+^CD43^+^CD93^+^CD23^-^CD21^-^ age-associated B-cells; F4/80^+^siglec f^+^ eosinophils; F4/80^H^CD11c^Int^ macrophages; F4/80^Int^CD11c^H^ dendritic cells and LyG6^+^ neutrophils.


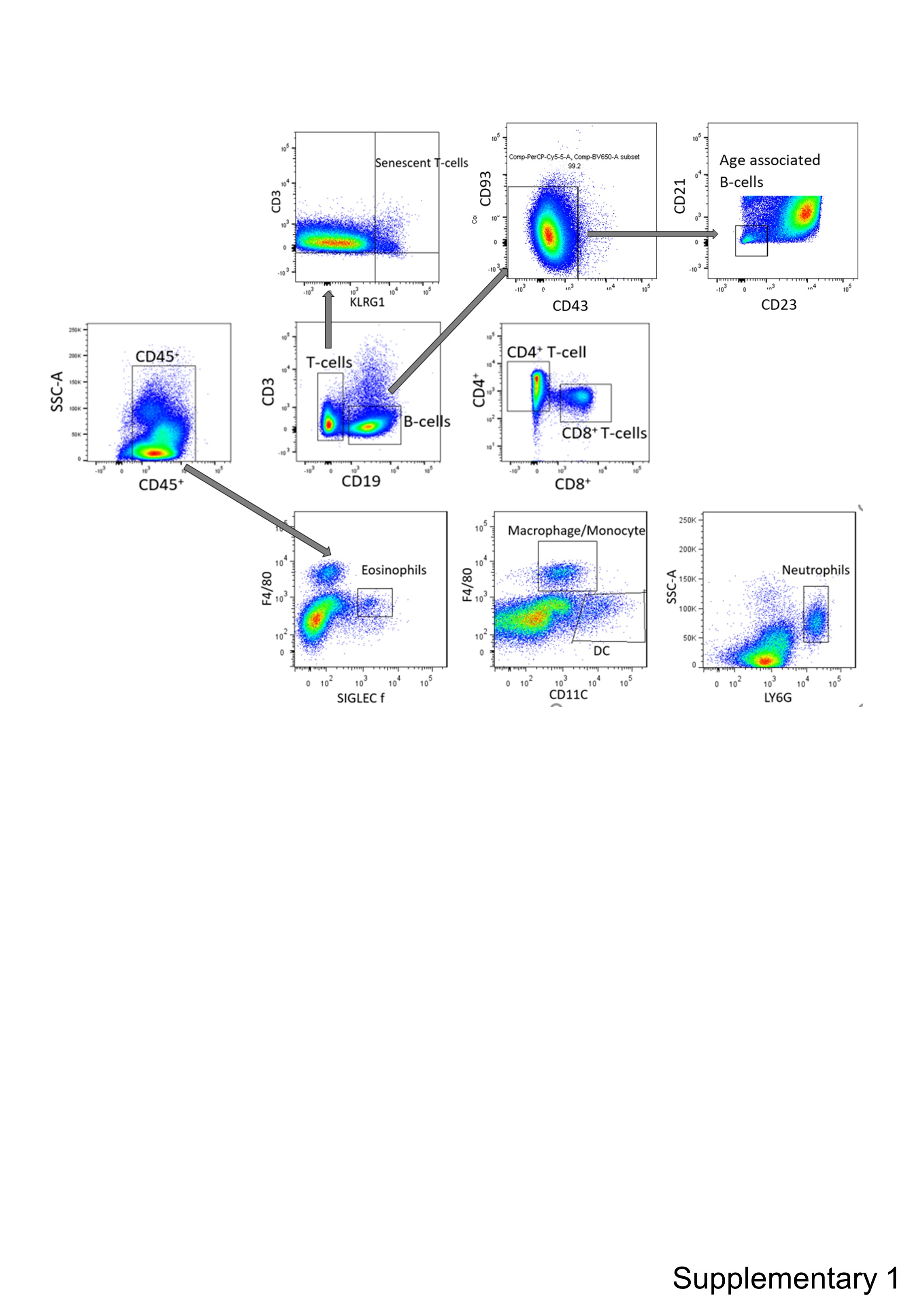


**Supplementary Figure 2 – PEPITEM treatment had no effect on metabolic changes associated with an obesogenic diet.** Mice were fed HFD for either **(A-B)** 6 or **(C-D)** 12 weeks and received 0.0822mg/week of PEPITEM or PBS as a control for either the full duration or for the last 6 weeks of the HFD. Body weight was assessed weekly for **(A)** 6 or **(C)** 12 weeks of HFD. Fasting blood glucose concentrations were measured at over 120 minutes in mice following **(B)** 6 or **(D)** 12 weeks of HFD. Data are mean ± SEM using (A-B) n = 5 or (C-D) n=14 mice per group from n = 1 independent experiment.


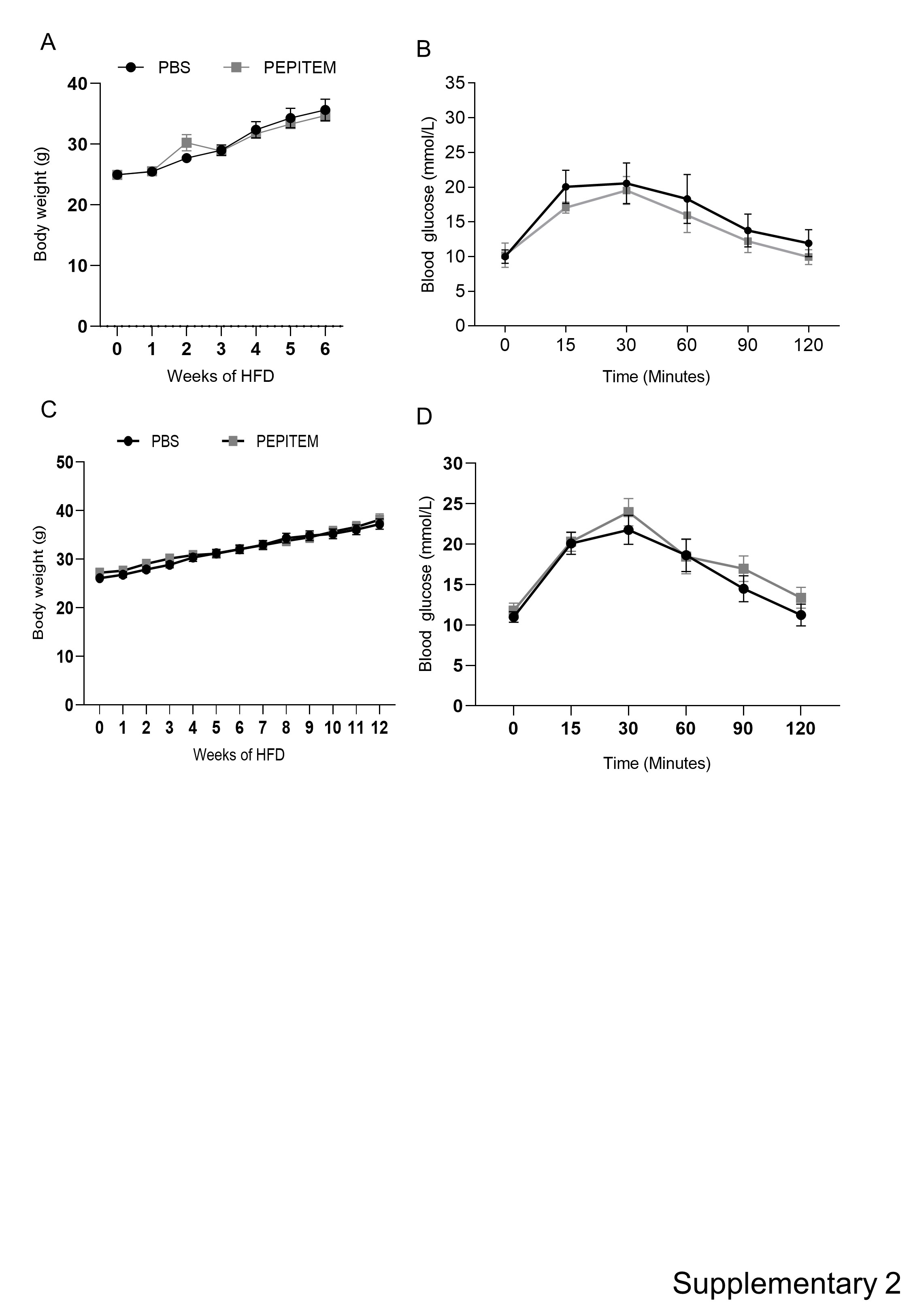


**Supplementary Table 1 – Quantification of other leukocyte subsets in mice on HFD for 6 weeks**

| **Leukocyte subset (Number)** | **Blood/ml** | | **Subcutaneous adipose**  **Tissue/g** | | **Spleen/g** | | **iLNs** | |
| --- | --- | --- | --- | --- | --- | --- | --- | --- |
|  | **PBS** | **PEPITEM** | **PBS** | **PEPITEM** | **PBS** | **PEPITEM** | **PBS** | **PEPITEM** |
| **CD45^+^ cell** | 116307±9038 | 108159±3169 | 28655±3311 | 30882±2353 | 37777237±  3795124 | 40914684±  3937809 | 137761±9322 | 168338±17547 |
| **B cell** | 37645±2128 | 44837±5613 | 1799±152.3 | 1859±100.9 | 18017788±  2422941 | 20549882±  3272735 | 26811±5889 | 24771±5804 |
| **ABC** | 5091±566.0 | 6895±834.7 | 1027±32.36 | 1007±53.37 | 1605879±  280099 | 1723345±  164344 | 241.3±49.36 | 294.2±56.49 |
| **T cell** | 35785±4418 | 40481±3827 | 14954±1491 | 16152±1673 | 5812659±  1284432 | 6841248±  1093347 | 99419±1338 | 105056±3970 |
| **CD4^+^ T cell** | 17686±2435 | 19895± 3260 | 9454±483.8 | 9483±417.3 | 3898541±  262349 | 3727943±  393053 | 30624±6550 | 34302±2347 |
| **CD8^+^ T cells** | 6764±1059 | 5868± 978.0 | 1484±145.0 | 1356±122.8 | 2658353±  341183 | 31795±1446 | 32976±760.9 | 34302±2347 |
| **T reg** | 140.0±5.055 | 138.7± 13.06 | 153.0±10.71 | 135.1±12.90 | 3299±452.8 | 3249±495.7 | 265.3±38.10 | 233.1±37.80 |
| **KLRG1 T cell** | 1762±363.0 | 1704±393.5 | 545.5±72.02 | 455.8±56.55 | 694010±  71650 | 689293±  71412 | 11427±712.8 | 11735±470.1 |
| **Monocyte/**  **Macrophage** | 14171±1812 | 15697± 1004 | 13260±1007 | 13691±1260 | 317833±  37650 | 307157±  37216 | 341.7±137.8 | 332.4±114.4 |
| **Eosinophil** | 1147±68.55 | 910.7±193.1 | 1643±99.73 | 1721±37.83 | 19308±3064 | 15359±3000 | 21.86±10.79 | 55.30±26.30 |
| **Neutrophil** | 8067±885.1 | 7605±1488 | 839.8±44.11 | 682.9±75.47 | 260316±  18831 | 223631±  12099 | 96.57±33.38 | 94.97±51.71 |

ABC = age-associated B-cells; iLN = inguinal lymph node; T reg = regulatory T cells.

Data are shown as mean ± SEM for n = 1 independent experiments, using n=14 mice per group.
